# Supplementary figures and images for: Metabolic Responses to Arsenite Exposure Regulated through Histidine Kinases PhoR and AioS in Agrobacterium tumefaciens 5A
Source: Microorganisms. 2020 Sep 2;8(9):1339. doi: 10.3390/microorganisms8091339 (PMC7565993; doi:10.3390/microorganisms8091339)

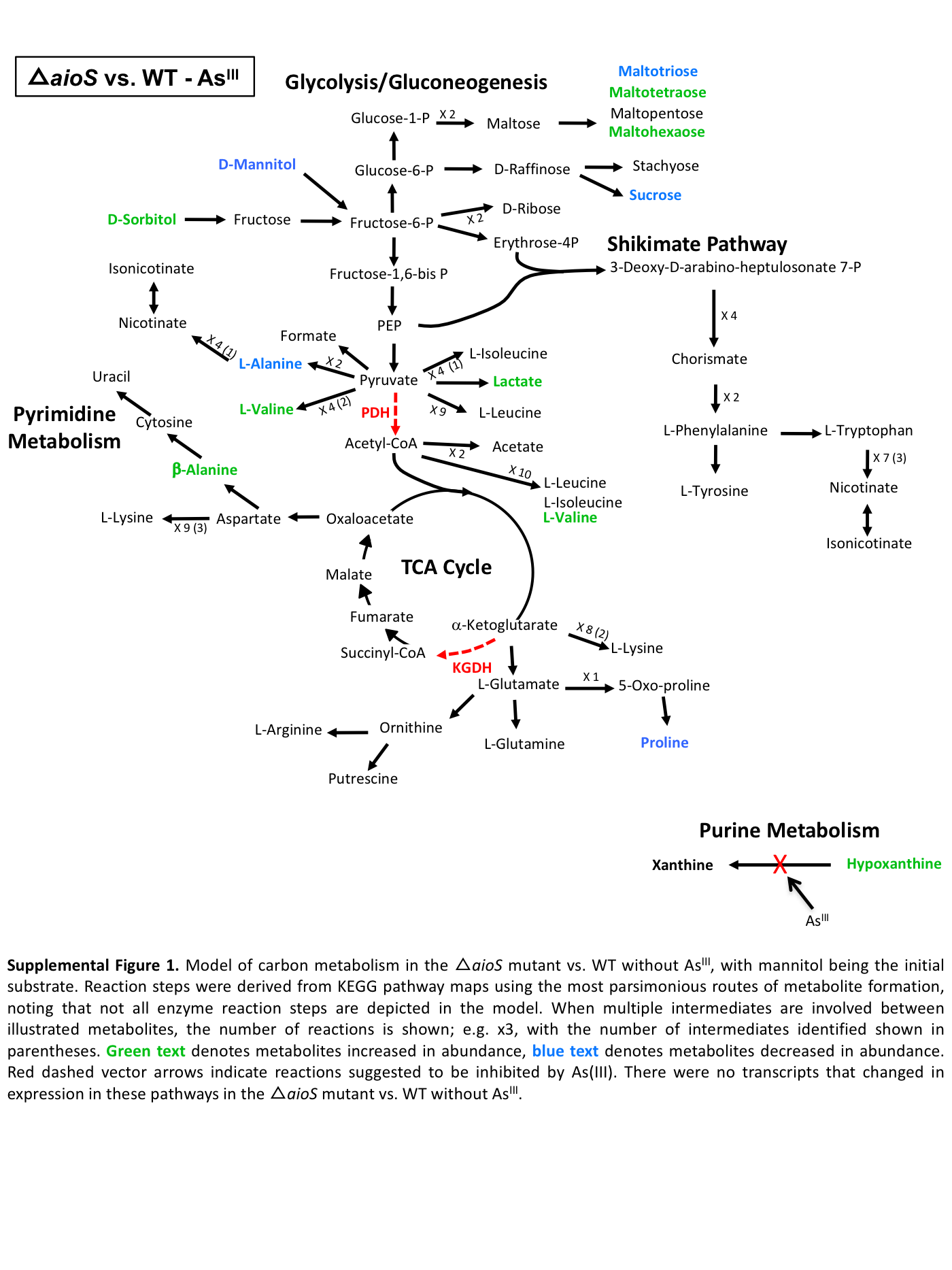

Supplement: Supplementary file 1 [file microorganisms-08-01339-s001.zip › Suppl-Material/Suppl-Figure-1.tiff]

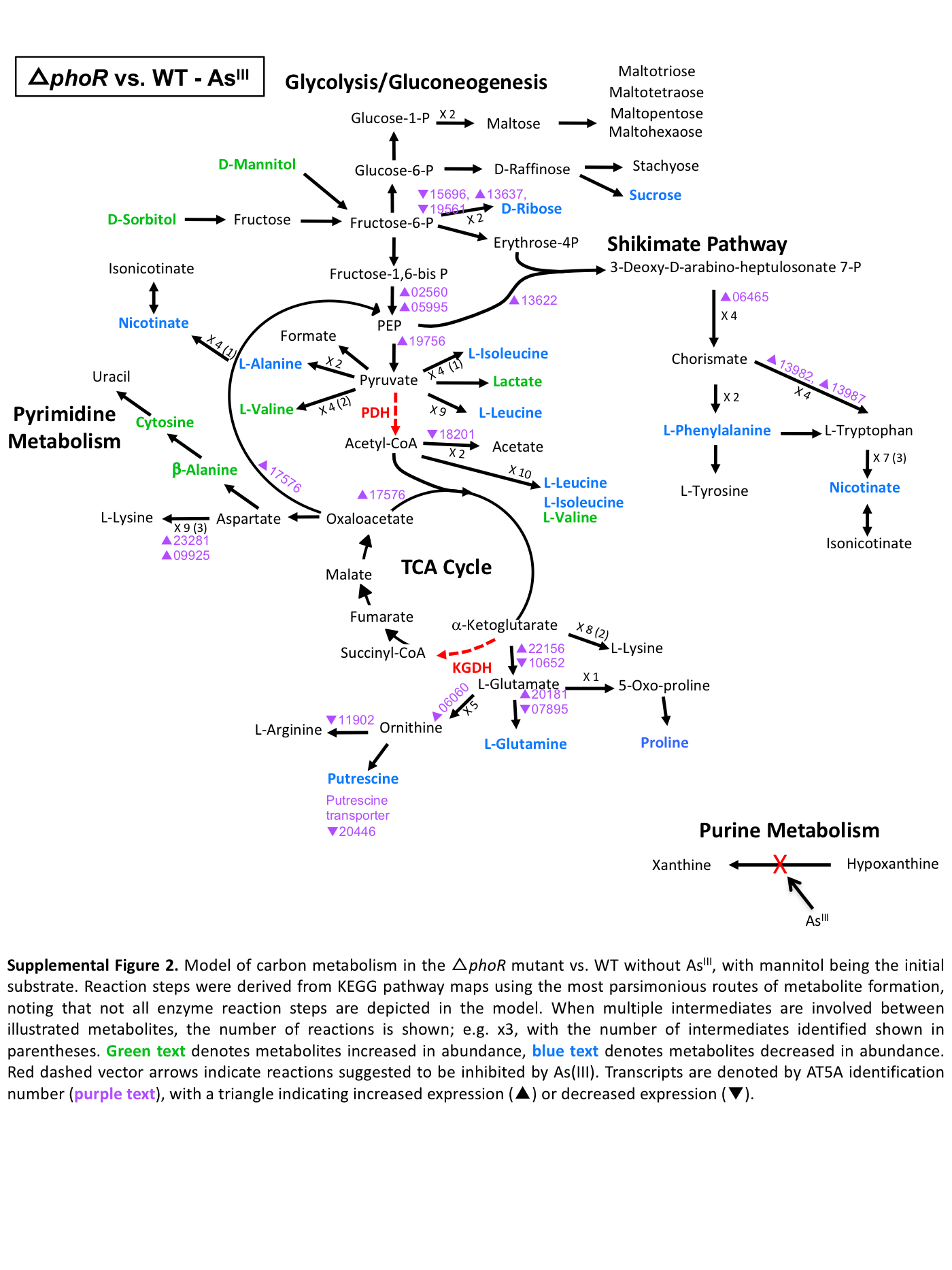

Supplement: Supplementary file 1 [file microorganisms-08-01339-s001.zip › Suppl-Material/Suppl-Figure-2.tiff]
